# Emerging analytical methods to characterize zeolite-based materials

**DOI:** 10.1093/nsr/nwac047

**Published:** 2022-03-12

**Authors:** Sophie H van Vreeswijk, Bert M Weckhuysen

**Affiliations:** Inorganic Chemistry and Catalysis, Debye Institute for Nanomaterials, Utrecht University, Utrecht 3584 CG, The Netherlands; Inorganic Chemistry and Catalysis, Debye Institute for Nanomaterials, Utrecht University, Utrecht 3584 CG, The Netherlands

**Keywords:** catalysis, zeolites, spectroscopy, microscopy, structure–composition–performance relationships

## Abstract

Zeolites and zeolitic materials are, through their use in numerous conventional and sustainable applications, very important to our daily lives, including to foster the necessary transition to a more circular society. The characterization of zeolite-based materials has a tremendous history and a great number of applications and properties of these materials have been discovered in the past decades. This review focuses on recently developed novel as well as more conventional techniques applied with the aim of better understanding zeolite-based materials. Recently explored analytical methods, e.g. atom probe tomography, scanning transmission X-ray microscopy, confocal fluorescence microscopy and photo-induced force microscopy, are discussed on their important contributions to the better understanding of zeolites as they mainly focus on the micro- to nanoscale chemical imaging and the revelation of structure–composition–performance relationships. Some other techniques have a long and established history, e.g. nuclear magnetic resonance, infrared, neutron scattering, electron microscopy and X-ray diffraction techniques, and have gone through increasing developments allowing the techniques to discover new and important features in zeolite-based materials. Additional to the increasing application of these methods, multiple techniques are nowadays used to study zeolites under working conditions (i.e. the *in situ*/*operando* mode of analysis) providing new insights in reaction and deactivation mechanisms.

## Teaser

The paper reviews the advanced, novel and emerging analytical methods to study zeolite properties and (catalytic) working behaviour.

## INTRODUCTION

Their unique characteristics and their wide variety of applications make zeolites one of the best-studied inorganic porous materials of the current time. Zeolite materials are used in everyday products, biomedical applications and to tackle multiple climate-changing problems as sustainable replacements for, for instance, the depletion of crude oil and plastic recycling as catalyst materials [1–3]. This great diversity of applications mainly finds its nature in the distinct characteristics of the zeolite-based materials. Zeolites possess acid sites, arising from their elemental composition and structure, which can act as active catalytic or ion-exchange centers [4]. Additionally, their defined porous structure makes them suitable for an even larger number of applications as they can be used as molecular sieves.

Zeolite research has a tremendous history from its discovery in 1756 [5]. The discovery of specific and unique zeolite properties were the basis for zeolite-based materials being used as functional materials in everyday processes. For instance, after the discovery of the acidic, and thereby catalytic, properties of the material, the impact of zeolite research increased enormously as the material was able to increase the oil-cracking efficiency and aid in solving climate problems (e.g. catalytic elimination of air pollutants, water purifications, biomass conversion, electrocatalytic fuel cells and the methanol-to-hydrocarbons (MTH) process) [2,4].

**Figure 1.**
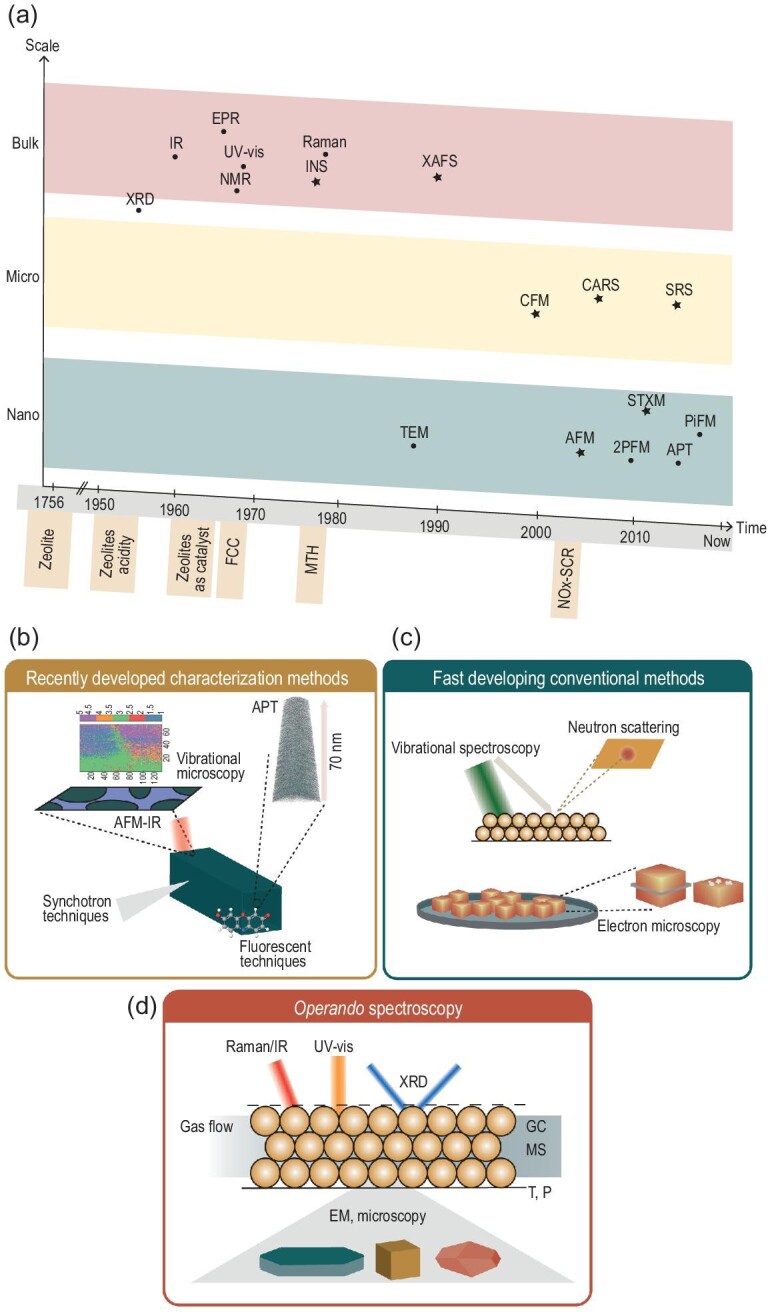
(a) Brief overview of some analytical techniques and milestones in zeolite chemistry using infrared (IR), X-ray diffraction (XRD), nuclear magnetic resonance (NMR), electron paramagnetic resonance (EPR), inelastic neutron scattering (INS), X-ray absorption fine structure (XAFS), ultraviolet-visable (UV-vis), coherent anti-stokes Raman scattering (CARS), confocal fluorescence microscopy (CFM), stimulated Raman spectroscopy (SRS), transmission electron microscopy (TEM), atomic force microscopy (AFM), scanning transmission X-ray microscopy (STXM), two-photon fluorescence microscopy (2PFM), photo-induced force microscopy (PiFM) and atom probe tomography (APT). (b–d) Schematic representation of the grouping of the techniques discussed in this review. Recently developed techniques are focused on new techniques to study zeolites while fast developing conventional methods are techniques that have been used in the zeolite characterization for a very long time but have emerged in their application to unravel zeolite properties over recent years. Some characterization techniques can also be used to study zeolites under working conditions (*operando* spectroscopy and microscopy), which gives insights into changes in the zeolite material or the mechanisms of the processes involving zeolite-based materials.

Conventional methods for characterizing zeolites, like X-ray diffraction (XRD), infrared (IR), UV-vis spectroscopy, Raman spectroscopy and nuclear magnetic resonance (NMR), were first applied to study zeolite materials from the 1950s to 1980s and were the basis of the discovery of the unique and fascinating properties of the zeolite-based materials resulting in the exploration of the acidic nature of the zeolites and its applications in catalysis. Before 1980, the techniques had mainly focused on revealing zeolite properties on the bulk scale. From 1980, the techniques developed to study (porous) materials slowly shifted to focus on the microscale and later on the nanoscale properties. This nanoscale property information allowed the further exploration of zeolites and their behavioral changes upon application. Moreover, both more conventional (i.e. developed analytical techniques between 1950 and 2000) and more recently introduced techniques are starting to be used to study zeolite-based materials under working conditions (i.e. *operando* spectroscopy and microscopy) resulting in tremendous breakthroughs in clarifying reaction and deactivation mechanisms. Figure 1a depicts an overview of the introduction of a selection of characterization techniques and their application for the development of structure–composition–performance relationships in zeolite science and technology.

As the investigation into zeolites properties and working conditions has such a great history of publications, it is impossible to include all the zeolite-characterization techniques in this review, nor is it possible to discuss all the important research performed in this field. Many other comprehensive reviews on zeolite characterization and reviews on specific analysis methods for zeolite characterization exist and we will therefore build on those and summarize their important findings. Zeolite characterization is a constantly evolving field and, as such, this review focuses on the emerging analytical methods of the last few years, divided into three subjects listed with the specific techniques referenced with previous specific key-reviews: (i) recently developed methods to study the materials (e.g. atom probe tomography (APT) [6,7], fluorescence microscopy techniques, such as confocal fluorescence microscopy (CFM) [8], photo-induced force microscopy (PiFM) [9] and synchrotron-based irradiation techniques, Fig. 1b); (ii) a selection of more conventional methods undergoing a revolution to be used in a novel way (e.g. Raman [10,11], neutron scattering (NS) [10,12,13], electron paramagnetic resonance (EPR) [14,15] and IR [10] spectroscopy, NMR [16], XRD [17] and electron microscopy (EM) [18–22], Fig. 1c); and (iii) techniques that are used to study zeolites under realistic working conditions (i.e. the *operando* characterization mode of operation, Fig. 1d). The recently developed analytical techniques we discuss are all focused on nanoscale chemical imaging or relations in the zeolite materials and these analytical techniques have thereby contributed to the further elemental understanding of the zeolite materials. The more conventional techniques undergoing revolution are focusing on imaging and bulk characterization with an innovative addition to improve their suitability for studying porous solids, such as zeolites. *Operando* characterization techniques help to understand how and why zeolites are changing under working conditions in real-life industrial-type applications. This includes the adsorption of molecules, as well as their catalytic performance in different processes. We do not aim to review the techniques one by one nor do we aim to give a detailed explanation about the working principles of each of the analytical techniques under study. Rather we focus on the technique's application to the field by highlighting some of the recent or most important research performed in this field and their vital outcomes, thereby hopefully inspiring newcomers in the field to further use these tools for the benefit of their own research or particular application.

## RECENTLY DEVELOPED METHODS: NANOSCALE IMAGING AND RELATIONS IN ZEOLITES

All analytical techniques that provide information at the nanoscale are either relatively novel or extremely relevant to the zeolite chemistry research. However, in this review, we have focused on a selection of techniques that, in our opinion, are the most novel or promising without aiming to be complete. Some analysis methods are already frequently applied to characterize zeolites; others we would like to bring attention to highly promising techniques as they have proven to be very useful in the analysis and exploration of other inorganic materials. Novel imaging and tomography techniques are often performed at synchrotron beam-line research centers. However, there are also some lab-based (tomography) techniques, which can be applied without a large dedicated energy source. Some techniques discussed in this section can also be used under *operando* conditions and will be discussed separately in ‘Zeolite characterization under working conditions: operando spectroscopy’ Section.

### Synchrotron-based microscopy

To obtain information with greater chemical spatial resolution, some analytical methods require a high-intensity beam source. These high energies can be generated by a synchrotron. In this section, we therefore discuss the most novel techniques recently developed for its application to zeolite characterization.

**Figure 2.**
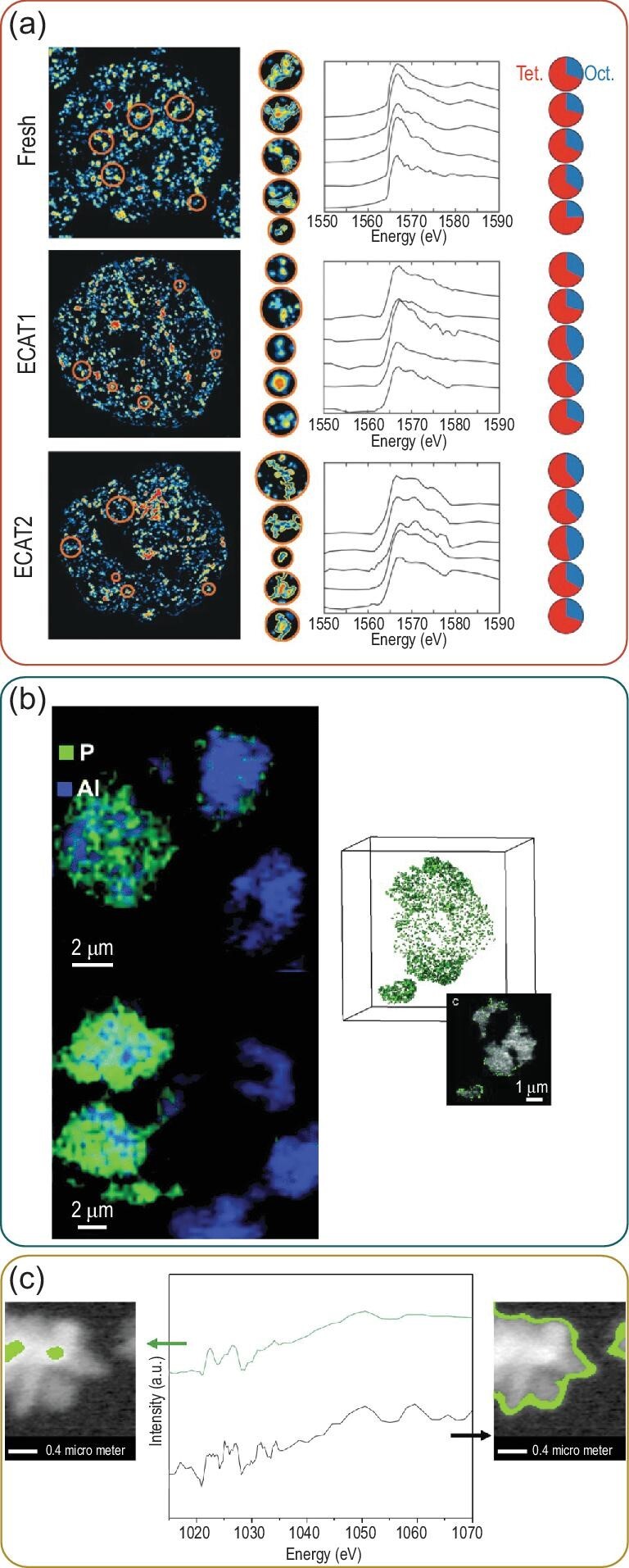
(a) Scanning transmission X-ray microscopy maps of La showing the individual zeolite domains with their corresponding X-ray absorption near-edge structure spectra allowing the determination of the distribution of the nature of Al: tetrahedral (red) and octahedral (blue). Figure adapted with permission from Ref. [24], Copyright (2016) Wiley-VCH. (b) Chemical maps of zeolites and their P distribution showing a higher concentration of phosphorus on the outer surface of the zeolite particle (green). Figures reproduced with permission from Ref. [27] from the Royal Society of Chemistry and from Ref. [28], Copyright (2014) Wiley-VCH. (c) STXM images of the pristine Zn-modified ZSM-5 particles indicating the middle and the outside of the catalyst particle showing their corresponding X-ray absorption near-edge structure spectra indicating a higher concentration of Zn (green area) in the inner pores of the pristine Zn–ZSM-5 zeolite. Figure is reproduced with permission from Ref. [30], Copyright (2019) Elsevier.

Aramburo *et al.* showed that scanning transmission X-ray microscopy (STXM) could be applied to zeolites and provided information about their physicochemical properties on the nanoscale [23]. This technique can combine the high chemical sensitivity of X-ray absorption spectroscopy (XAS) with the spatial resolution of microscopic techniques (≤10–70 nm) resulting in images with nanoscale resolution containing chemical information [23,11,24]. Therefore, this technique has mainly been used to obtain information about the zeolite framework element distributions in, for instance, dealuminated or aged zeolite samples. For fluid cracking catalyst (FCC) materials, it was found that using La as a quantitative marker for the zeolite regions, upon aging in their catalytic process, a heterogeneous dealumination process takes place (on a single catalyst particle level). The percentage frequency of tetrahedral and octahedral Al in the zeolite domains, found with Al K-edge X-ray absorption near-edge structure (XANES) spectroscopy, changed heterogeneously with aging showing the technique's ability to study elemental distributions in industrial relevant materials like FCC particles (Fig. 2a) [24]. Using the same technique, for SAPO-34 it was found that, among other things, the XANES spectra were more defined in the pristine samples compared to the steamed samples, indicating that this treatment is decreasing, besides the number of acid sites, the crystallinity of the material [25]. The effect of promoters and binders on the performance of zeolites or on the enhancement of their thermal stability can also be studied with this technique. For instance, phosphatation of zeolites by the formation of a AlPO_4_ interaction enhances the hydrothermal stability of zeolites. With STXM imaging, both intra- and inter-particle heterogeneities were found with a higher concentration of phosphorus closer to or on the outer surface of the zeolite particles (Fig. 2b) [26–29]. Zeolites can also be modified by introducing metals to enhance their performance in catalytic reactions. Zn-modified zeolites are in this way used as catalysts to convert small molecules, such as methanol, to aromatic molecules [30]. He *et al.* discovered an inhomogeneous distribution of the introduced zinc with STXM analysis methods, which was dependent on the reaction progression. In the fresh catalyst, the Zn concentration was much higher in the inner pores, whilst during reaction, the Zn redistributed to the outer surface of the zeolite crystal (Fig. 2c) [30].

### Lab-based imaging and chemical mapping methods

Some techniques can provide (3D) nanoscale chemical information without the need for a high energy source, which allows these techniques to be used in general laboratories.

**Figure 3.**
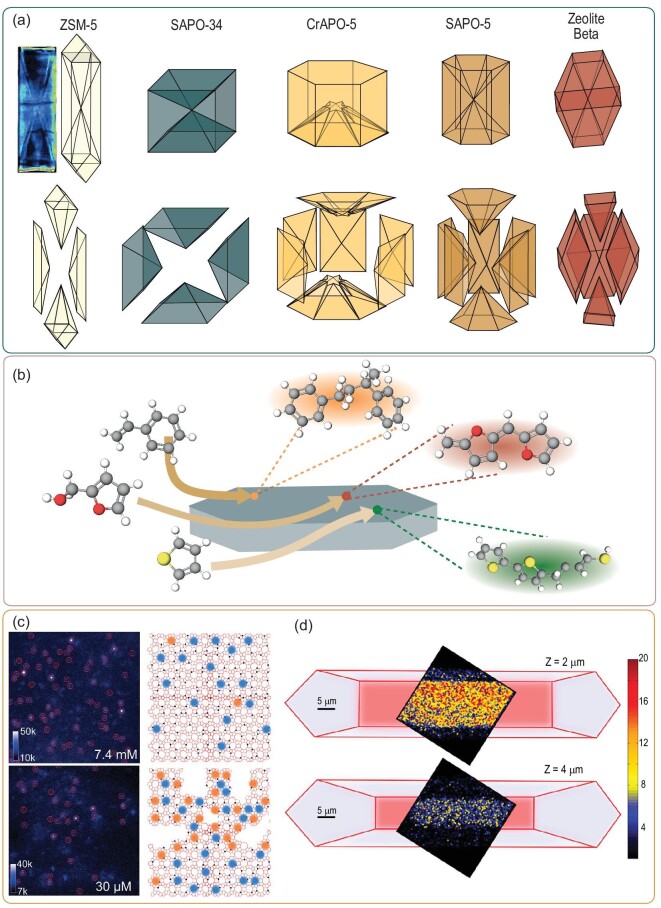
(a) Examples of intergrowth structures found using confocal fluorescence microscopy (CFM) (template removal and probe molecule studies) and one example of a confocal fluorescence image of zeolite ZSM-5. Figures adapted and modified with permission from Ref. [31], Copyright (2007), Wiley-VCH and [36], Copyright (2017) Wiley-VCH. (b) Schematic representation of examples of single-molecule probe reactions with zeolites, modified from Ref. [8], (2021) Springer Nature [39,38]. (c) Single-molecule maps of parent zeolite ZSM-5 (above) and steamed zeolite ZSM-5 (below) with different solvent-to-4-methoxystyrene ratios. The single fluorescence events are assigned with red circles indicating the efficiency of the localization algorithm. Steamed zeolite ZSM-5 allowed much larger fluorescent molecules to form (trimeric carbocations, orange dots) than in the parent zeolite ZSM-5 (dimeric carbocations, blue dots) due to the formation of defects in the zeolite structure. Figure adapted with permission from Ref. [39], Copyright (2016) American Chemical Society. (d) Single-molecule FM maps with ZSM-5 show different activity in different places of the zeolite crystal. Figure adapted with permission from Ref. [40], Copyright (2015) American Chemical Society.

One of those lab-based techniques, which has developed impressively in recent years, is fluorescence microscopy (FM). Probe molecules can be used to visualize the pore networks to reveal the internal building blocks of a zeolite crystal. These fundamental insights can provide information on the structure–function relations in these materials. Some zeolite structures do not possess cages and pores large enough for hosting organic probe molecules. In this case, coke molecules, residues from, for instance, the MTH reaction and the ‘template-removal approach’ can be used to study the structure–function relations in these materials [31–33]. The template ions (e.g. tetrapropylammonium), used in the zeolite synthesis, are hydrocarbon-containing molecules, which are, upon the template-removal process, increasingly fluorescent so that these template molecules can essentially be used as fluorescent probe molecules to study the internal architecture of the zeolite crystals upon de-templating [34]. Karwacki *et al.* found that while partially decomposing the template molecules, the diffusion was hindered by diffusion barriers leading to the clear visualization of the internal intergrowth structure of zeolite ZSM-5 by the use of CFM [35]. Such an intergrowth structure was not only observed for large zeolite ZSM-5 crystals, but has also been observed for other zeolite frameworks (i.e. CHA, BEA and AFI) and an overview of different intergrowth structures found with CFM is depicted in Fig. 3a [31,36,37]. The ability of this technique to analyse fluorescent hydrocarbon intermediate and deactivating components in multiple reactions makes the techniques very suitable for studying materials under working conditions as discussed in ‘Zeolite characterization under working conditions: operando spectroscopy’ Section. Probe molecules and FM can also be used to study the zeolite local activity using single-molecule localization microscopy (SMLM) [8]. Roeffaers and co-workers have applied single-molecule FM techniques to assess the organic reactivity of zeolite materials in ZSM-5 and ZSM-22. The new nanometer accuracy by stochastic chemical reactions (NASCA) microscopy technique is based on the formation of a fluorescence molecule on the acid/active sites of the zeolite (turnover activities) [38]. In Fig. 3b, the technique is schematically illustrated and some examples of the formation of fluorescent molecules within zeolites are depicted. Ristanović *et al.* found that differences in measured turnover activities depend on the differences in 3D aluminum distributions upon zeolite steaming (Fig. 3c) and on the pore network (straight and sinusoidal) within one crystal of zeolite ZSM-5 (Fig. 3d). They showed that steaming leads to the formation of defects in the structure allowing the formation of larger fluorescent molecules such as trimeric carbocations compared to the parent (non-steamed) zeolite ZSM-5 material [39,40]. Actual single-molecule tracking experiments to study diffusion, previously demonstrated to be working for other inorganic materials, were performed by our group. This approach was used to track the diffusion of single molecules in the zeolite channels using single orientated zeolite ZSM-5 thin films, revealing a strong diffusion heterogeneity and a great dependency of the crystallographic axis resulting in a diffusion anisotropy [41].

**Figure 4.**
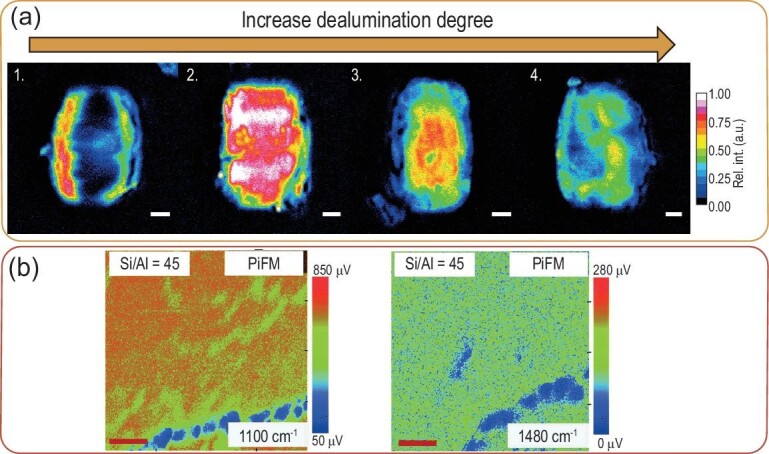
(a) The evolution of the stimulated Raman-scattering microscopy images upon further dealumination of zeolite mordenite showing first an increase in accessibility and then a decrease in Al and active sites. Scale bars represent 2 μm. Figure reprinted with permission from Ref. [43], Copyright (2020) American Chemical Society. (b) PiFM images taken at two different wavelengths to amplify two different elemental compositions: 1100 cm^–1^ (framework vibrations) and 1480 cm^–1^ (hydrocarbons vibrations). The scale bars represent 1 μm. Figures obtained from Ref. [44] with permission from the Royal Society of Chemistry.

Some groups use FM for correlative studies combining, for instance, scanning electron microscopy (SEM) or stimulated Raman scattering (SRS) microscopy [11,42]. The latter technique increases the Raman signal sensitivity by using picosecond Stokes and pump lasers [10]. In the SRS analysis techniques, probe molecules can be used to obtain information about the acid-site distribution on the zeolite single-crystal level. Roeffaers and colleagues used nitrile and pyridine probes to study small-pore mordenite crystals [11,43]. They found that the distribution of Brønsted acid sites within zeolites can be monitored and resolved using the ring breathing mode of pyridinium ions (1006 cm^–1^) in SRS microscopy, as long as the pores of the zeolite are large enough for the diffusion of this molecule. They studied the effect of dealumination (SRS images are depicted in Fig. 4a) on the crystal structure of mordenite and found that the zeolite structure and aluminum distribution are strongly dependent on the degree of dealumination. The small pores inside zeolite mordenite are not affected by the treatment and pyridine does not diffuse in at lower degrees of dealumination (Fig. 4a-1). With a slightly higher degree of dealumination, the pyridine can already find its way to the inside of the crystal (Fig. 4a-2). Larger degrees of dealumination show that the intensity is decreasing due to the lower content of aluminum and thereby the removal of the active sites in the zeolite (Fig. 4a-3–4). Additionally, samples from the same batch show significant inter- and intraparticle heterogeneity of the acid and thereby the active centers of the material. However, the diffusion of the pyridine molecules is the limiting factor for the application of this method to other zeolite frameworks [43]. Consequently, other techniques can help to explore the zeolite element distribution on the nanoscale. For example, Fu *et al.* found that PiFM can also be applied to study the growth mechanism of zeolite thin films while investigating the effect of different structure-directing agents and their influence on the structure–performance relations in the MTH reaction (Fig. 4b). Even though a heterogeneous distribution of Al was found on the surface, the hydrocarbon distribution was found to be homogeneous. This was attributed to the long reaction time, resulting in a high number of coke molecules (large amount of coke) [44]. By combining Raman spectroscopy and atomic force microscopy (AFM), a promising technique was developed, namely tip-enhanced Raman spectroscopy (TERS). This combines the capabilities of analysing the chemical bonding of zeolitic materials (Raman) with very low spatial resolution (AFM). As we are not aware of any publications regarding zeolitic materials and TERS, we would like to highlight this technique as very promising for further revelation of structure–composition–performance relations [10,45].

**Figure 5.**
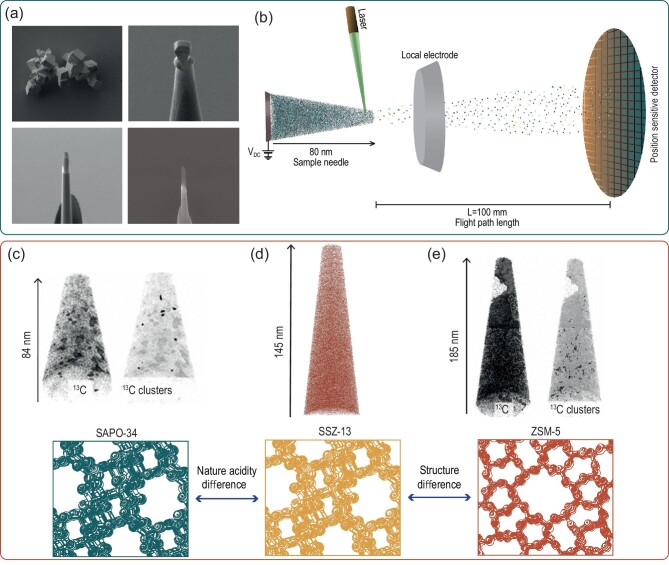
(a) SEM images of the needle preparation from zeolite HSSZ-13 crystals with FIB-milling techniques. Figures adapted from Ref. [55] with permission from the Royal Society of Chemistry. (b) Schematic representation of the atom probe tomography techniques. Figure redrawn with permission from Ref. [46], Copyright (2018) Wiley-VCH and a ‘real reconstructed’ needle is used. (c) Schematic representation of SAPO-34 crystal structure and the carbon-ion maps corresponding to this structure with the carbon clusters found in this single needle. Ion maps reproduced from Ref. [53], Copyright (2018) American Chemical Society. (d) Schematic representation of the SSZ-13 structure and the carbon-ion maps without cluster formation. Ion maps obtained from Ref. [55] with permission from the Royal Society of Chemistry. (e) Schematic representation of the zeolite ZSM-5 structure and carbon-ion maps with the carbon clusters found and carbon-depleted areas. Ion maps reproduced with permission from Ref. [53], Copyright (2018) American Chemical Society.

APT can be used to study zeolites on a subnanometer scale as, in contrast to other techniques, APT has the advantage that it can easily differentiate between the zeolite framework elements (very similar z-contrast elements Si, Al, O) [46]. Some recent studies of the last 6 years have shown the great possibilities of this technique for studying zeolite properties [47–50]. APT is based on ion-by-ion field evaporation, which is caused by a combined electric field and a pulsed laser applied on a needle-shaped material (prepared with FIB-milling techniques) as shown in Fig. 5a [51]. Identification of the ions is performed by mass spectrometry (MS) using time-of-flight and the position of the ions is determined using a position-sensitive detector allowing 3D reconstructions (a schematic representation of the set-up is depicted in Fig. 5b) [46]. Nanoscale heterogeneities of the framework elements, introduced metals for enhanced catalytic activity and deactivating compounds (coke) can be found and analysed with this technique. For instance, with this method, the distribution of Cu in automotive catalysts Cu-SSZ-13 and Cu-ZSM-5 was identified and compared before and after reaction, explaining the greater catalytic performance of Cu-SSZ-13 over Cu-ZSM-5 zeolites due to relations on the nanoscale. The copper is heterogeneously distributed in these samples and Cu clusters of just a few nm can be visualized in 3D [47]. This technique has also been correlated to STXM, which further contributed to the micro-to-nanoscale insights in the selective catalytic reduction (SCR) process [52]. Additionally, carbon clusters, residues of the MTH of just a few nanometers, were isolated in ZSM-5 and SAPO-34 allowing the spatial determination of coke molecules on the nanoscale [53,54]. These carbon clusters were also linked to the Al concentration and location in the zeolite from micro to nanoscale [54]. Very recent studies also compare these results to the SSZ-13 structure in which the carbon is much more homogeneously distributed—something that can be linked to the bulk coking behavior and the zeolite properties [55]. Identification of these clusters together with similar isolation methods allows the comparison of carbon clusters between different zeolite frameworks and is shown in Fig. 5d–f.

## REVOLUTIONIZING CONVENTIONAL METHODS: NEW INSIGHTS

Some analysis techniques were proven to be suitable for zeolite characterization decades ago and are, in essence, not specified as ‘novel’. In this part, we would like to emphasize some of these important techniques that have been undergoing a revolution in their use for zeolite characterization over the last few years. Most of these techniques have been able to unravel some important questions regarding zeolites and their applications to an extent that these techniques were not capable of in the past.

**Figure 6.**
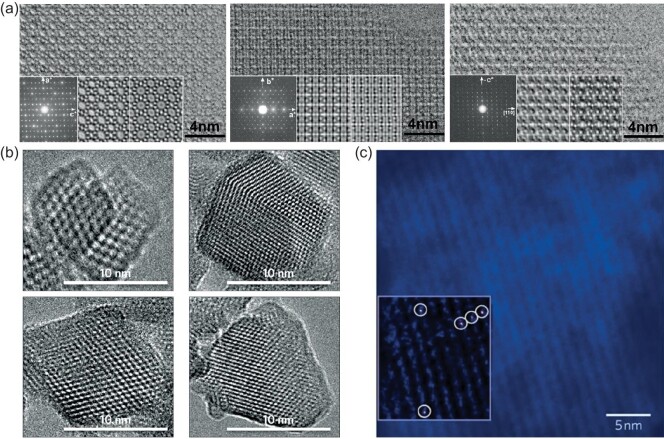
Some examples of the applications of electron microscopy to analyse zeolites. (a) Using transmission electron microscopy (TEM) (and X-ray diffraction (XRD) techniques), the structure of zeolite TNU-9 has been unraveled and the TEM images are shown along different axes of the crystal: [010], [001] [110], from left to right together with their corresponding electron-diffraction patterns. Figures adapted with permission from Ref. [56], Copyright (2006) Nature Publishing Group*.* (b) Electron microscopy has been used to analyse nanosized zeolite crystals showing here TEM images of zeolite Y with a crystal size of ∼10 nm. Figures adapted with permission from Ref. [69], Copyright (2015) Nature Publishing Group. (c) High-angle annular dark-field scanning transmission electron microscopy (HAADF-STEM) showing individual Ir ions anchored to the zeolite structure. Figure reproduced with permission from Ref. [72], Copyright (2010) Nature Publishing Group.

The characterization of zeolites using EM has been established already for a long time. However, over the last decades, the spatial resolution, elemental sensitivity as well as its suitability to analyse zeolite materials has been developed very fast [19]. With transmission electron microscopy (TEM), structural properties, morphology and particle sizes of the zeolites under study can be determined. Combining this technique with electron energy loss spectroscopy (EELS) or energy dispersive X-ray spectroscopy (EDS) allows this technique to be used to investigate the chemical compositions, both qualitatively and quantitatively [21]. High-resolution transmission electron microscopy (HRTEM) has been used for zeolite characterization since the 1980s as many zeolite structures can be directly visualized [20,18]. However, to obtain structural information, EM is now often being used in combination with XRD and electron diffraction (ED). This combination of techniques has been extremely important for the invention of new zeolitic materials [19,20]. In 2006, Terasaki and co-workers revealed the complete structure of TNU-9 (an extremely complex system) by this combination of techniques (HRTEM, ED and XRD) [56]. Figure 6a shows the HRTEM images taken from different axes along the crystal plane together with the corresponding ED patterns. Other important examples of zeotype materials for which the structure was determined in the last 15 years using the combination of techniques are SSZ-74 and IM-5, ITQ-37 and ITQ-39 [57–60]. More recently, several structures were solved using 3D ED methods that commonly make use of a rotating goniometer, e.g. ITQ-54, ITQ-56, PKU-16 and ITQ-58 [61–67].

Besides the ability to resolve complete crystal structures, EM can also be used to study the morphology of zeolite materials and follow their changes upon, for instance, the synthesis procedure [20,68]. Additionally, the crystal size of the zeolite does play an important role in the catalysis. Small crystals show an improved lifetime compared to larger crystals of the same material in multiple catalytic reactions. EM can be used to study these smaller, sometimes nanosized, zeolite crystals (Fig. 6b) [21,69]. Moreover, since zeolites are widely used because of their ion-exchange capabilities and the introduction of metal nanoparticles can provide the material with bifunctional properties, information about cations and nanoparticles located within the pores and cages of the zeolites is desired [70,71]. Ortalan *et al.* showed that high-angle annular dark-field scanning transmission electron microscopy (HAADF-STEM) can be used to image iridium clusters and mononuclear species within the cages of zeolite Y (FAU) [72]. Figure 6c depicts the individual Ir ions anchored to the zeolite structure. Lately, scanning transmission electron microscopy techniques (STEM) have even been applied to study and ‘image’ single molecules and other atoms in zeolite channels [73–75].

**Figure 7.**
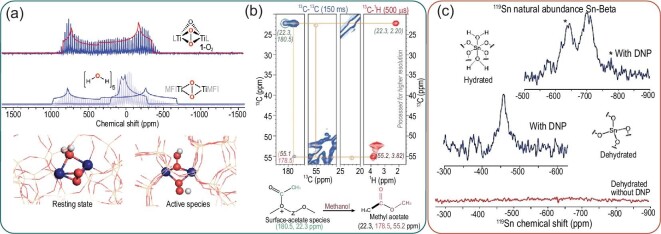
(a) Experimental ^17^O–NMR spectra and the corresponding calculated spectra for peroxo species on a homogeneous epoxidation catalyst and Ti–MFI showing very similar trends for calculated and experimental spectra supporting the proposition of this work for dinuclear Ti sites. Figures obtained with permission from Ref. [89], Copyright (2020) Springer Nature Limited. (b) 2D ^13^C–^13^C ^13^C–^1^H SSNMR spectra of the formed surface-acetate and methyl-acetate species on SAPO-34 and their corresponding chemical shifts to illustrate the principle. Figures reproduced with permission from Ref. [92], Copyright (2016) Wiley-VCH. (c) DNP-enhanced ^119^Sn NMR spectra (blue) of the hydrated and dehydrated Sn-Beta zeolite acquired for 18 and 21 h and normal ^119^Sn NMR spectra without DNP (red) acquired for 246 h. This shows that the sensitivity is enormously enhanced with the use for DNP and that this technique allows the analysis of ^119^Sn without isotopic enrichment experiments. Figure copied with permission from Ref. [97], Copyright (2014) American Chemical Society.

Solid-state nuclear magnetic resonance (SSNMR) can be used to define structural and chemical properties within zeolites. This analysis tool is predominantly used to study the acid sites within the zeolite materials [76]. Probe molecules can be used to quantitatively and qualitatively study the type, Lewis- or Brønsted acid sites (LAS/BAS), density and strength of the acid sites, and SSNMR also provides a way to look into the local environment of the acid sites [77–79]. SSNMR can also be used to study the effect of the introduction of other heteroatoms (e.g. Ga, Ge and B) to the framework and can be applied to contribute to resolve the crystal structure [16,77,80]. ^1^H–NMR can be used to study the BAS and LAS as they contain hydroxyl groups arising from, for instance, SiOH and AlOH [81,82]. In the same way, ^29^Si–NMR can be used to study the geometry of the zeolite and ^27^Al–NMR can be used to obtain information about the orientation of the aluminum atoms and thereby the active centers [81]. The different chemical shift experiments can also be applied in a correlative manner, improving the suitability of the SSNMR to the zeolite chemistry field as more structural and connectivity information can be obtained. The different chemical shift experiments can also be applied in parallel as the groups of Lercher and van Bokhoven, among others, have done to study the acid-site distribution changes (Al orientation in zeolites) and its effect on the zeolite framework [83,84]. 2D or double quantum (DQ) NMR is a method for studying atom proximities inside a zeolite catalyst and to investigate, for instance, dealumination (when using ^27^Al-^27^Al SSNMR) [85,86]. Additionally, the synergy between active sites in metal-containing zeolites can be studied with 2D double-resonance SSNMR experiments, especially when proton-detected methods are used as the metal–hydroxyl groups can be identified [87]. Double-resonance experiments allow the further revelation of these synergies as they are measuring the dipole–dipole interactions of close nuclei [85,87]. In this way, Deng and co-workers, recently used ^1^H-^95^Mo SSNMR spectroscopy to elucidate on the active sites in the methane dehydroaromatization (MDA) over Mo–ZSM-5 catalysts. They have hereby established proof of acidic proton–Mo dual sites. The combination with 2D ^1^H–^1^H even allowed the investigation of olefins as reaction intermediates [79]. Isotopic enrichment of zeolites with, for example, ^17^O has also been applied as the binding of ions to oxygen atoms in the zeolite framework is of great interest [88]. Gordon *et al.* proposed a new insight into the epoxidation of olefins by titanium silicalite-1 (TS-1) by studying the interaction with H_2_^17^O_2_ making use of ^17^O–NMR spectroscopy, density functional theory (DFT) calculations and other complementary spectroscopic techniques. They proposed that the Ti was incorporated into the framework of silicalite-1 forming dinuclear sites, as opposed to the mononuclear sites as proposed in earlier studies, explaining their specific catalytic activity [89]. Figure 7a shows that the calculated and experimental ^17^O–NMR spectra are matching, supporting the conclusion of this work that Ti exists as dinuclear sites within TS-1. Yang *et al.* recently clarified the framework characteristic changes and explained the effect of water on the stability of SAPO-34 catalyst upon reaction–regeneration in the MTH process by the use of NMR spectroscopy [90]. In the previously explained cases, the mechanism of a catalytic reaction has been explained by the catalyst properties, but SSNMR can also be used to study the catalytic reaction by analysing the reaction product residues in the zeolite, e.g. hydrocarbons residues [91]. With the use of SSNMR, Chowdhury *et al.* contributed to unraveling the initial carbon–carbon coupling in the MTH reaction by looking into the reacted hydrocarbon atoms using 2D SSNMR correlation experiments (^1^H-^13^C-NMR cross polarization and ^13^C-NMR direct excitation) on ^13^C-enriched coked samples. They provided proof of the initial carbon–carbon coupling process by identifying surface-bound acetate species, methyl acetate and dimethoxymethane [92]. Figure 7b shows the 2D SSNMR spectra of the SAPO-34 zoomed in on the contribution of surface acetate species and methyl acetate to illustrate the principle of the method. Later, they also proved, using a similar approach, that the initial formed species have a great influence on the formation of later reaction intermediates [93]. A similar method to study reaction products and intermediates was used by Román-Leshkov and co-workers. They reacted ^13^C-enriched glucose to study the epimerization reaction mechanism over zeolite Sn-Beta using ^13^C-NMR [94].

One of the drawbacks of NMR spectroscopy is that often the concentration of active nuclei can be very low, resulting in long measuring times. Dynamic nuclear polarization (DNP) methods can help to overcome these problems by increasing the NMR sensitivity and simultaneously avoiding the high costs of isotopic element enrichment, allowing the technique to be used for materials in which isotopic enrichment is not practical (industrial catalysts) [95,96]. In one of the earlier research performed using this method, zeolite Sn-Beta was studied. As the natural abundance of ^119^Sn is quite low and the loading of the metal in the structure is not very high, conventional NMR analysis is limited. High-quality data were much quicker to obtain using DNP and information on the active state of the Sn sites could be determined (Fig. 7c) [97,98]. Moreover, this technique's ability of enhancing the signals from the surfaces can also be applied to study oxygen atoms (^17^O) and thereby the acid sites of the zeolites. Using this approach, Perras *et al.* showed that pyridine can be used to identify the O–H distance and thereby the acidic nature of different groups within heterogeneous catalysts [99]. As many zeolite-catalysed reactions concern the formation hydrocarbon molecules and coke, the application of DNP has been proven in recent studies to be very useful in understanding the coking mechanism of not only zeolites, but also FCC particles [100–102].

**Figure 8.**
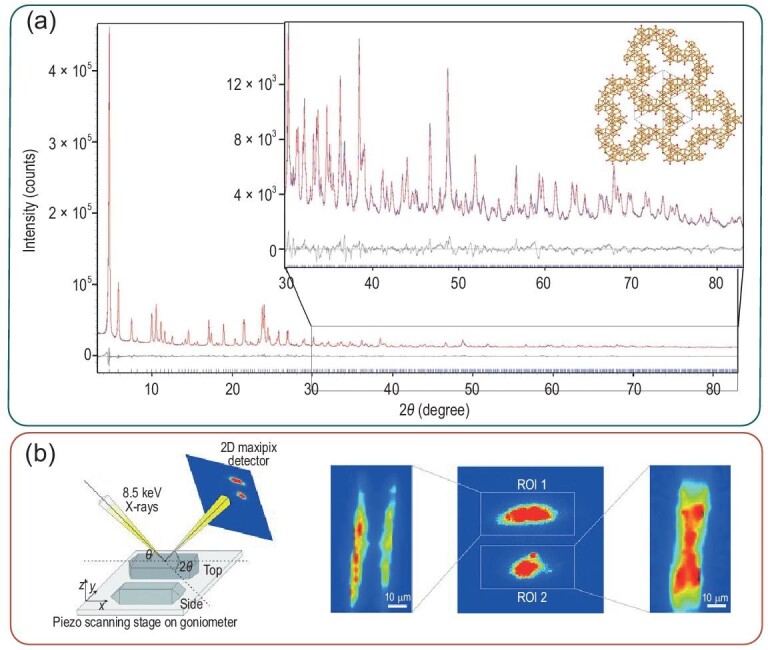
Examples of the use of X-ray diffraction (XRD) in the zeolite chemistry. (a) Powder XRD patterns (blue) taken to solve the structure of ITQ-37 zeolites using the calculated (red) Rietveld refinement and the differences between measured and calculated in black and a structural image of a part of the structure down the [111] direction. Figures reproduced with permission from Ref. [59], Copyright (2009) Macmillan Publishers Limited. (b) Schematic representation of the experimental set-up of micro X-ray diffraction crystallography imaging using a micro-focused X-ray beam moving the sample under the beam with a piezo stage to obtain spatial information. Additionally, some examples showing the diffraction response (diffraction intensity maps) in the region of interest (ROI) in a zeolite ZSM-5 coffin-shaped crystal. Pictures obtained with permission from Ref. [103], Copyright (2013) Wiley-VCH.

A very often used technique to define the crystal structure of zeolites requires single-crystal XRD or, sometimes, X-ray (powder) diffraction (PXRD) [62]. To be able to accurately define the crystal-structure properties and parameters of zeolitic powders, and thus the channel dimensions by refinements, it is often essential to have high-quality data that can be generated by high-intensity X-ray sources obtained at synchrotron beam lines (SXRD) [17]. The advances in SXRD have led to the higher use of PXRD to define the crystal structure from powders rather than single-crystal XRD facilitating the analysis of many industrial relevant catalysts. The structure of ITQ-37, a mesoporous germanosilicate, has been determined by the use of PXRD (lab-based) and ED. XRD patterns can be resolved by applying Rietveld refinement, allowing the definition of the lattice parameters of the zeolite structure [59]. Figure 8a shows an example of such a Rietveld refinement helping to resolve the crystal structure of the ITQ-37 zeolite framework. XRD can also be used to obtain spatially resolved information. Micro X-ray diffraction (μ-XRD) uses a micro-focused beam to detect diffraction peaks in a spatial region of interest. A schematic representation of the working principle/set-up is presented in Fig. 8b [103]. Ristanović *et al.* were among the first to use this crystallographic technique on zeolite crystals. They crystallographically mapped the different Al concentrations in large coffin-shaped zeolite ZSM-5 crystals and provided further insights into the crystallographic properties of the subunits of this model zeolite material [103]. Two examples of diffraction response on a 2D X-ray pixel detector are depicted in Fig. 8b. SXRD can also be used to follow the adsorption of molecules onto the zeolite framework, additionally allowing the use of adsorption molecules as probe molecules to study the zeolite properties. Lo *et al.* applied this technique in combination with Rietveld refinement analysis to study the BAS and LAS in HZSM-5 and HUSY by using DMF and Furan as probe molecules [104]. Chen *et al.* recently studied the adsorption of lysine, which stereospecifically (l/d) binds to the ZSM-5 zeolite [105]. Pair distribution function (PDF) analysis can be applied to obtain information on the short-range structure, with atomic distances that are challenging to determine using other techniques [106,107]. Using PDF, the structural evolution upon zeolite formation (i.e. synthesis and characterization) can be followed [106]. Normally, PDF analysis is limited in the use of synchrotron hard X-ray or neutron sources as high signal-to-noise ratios are required [108]. Another important technique in the zeolite-characterization research field using X-rays is XAS, which is very useful for instances in which the size domains are below the limits of XRD. This analysis method has recently been used to obtain information about zeolite materials under working conditions and will therefore be further discussed in ‘Zeolite characterization under working conditions: *operando* spectroscopy’ Section. Small-angle X-ray scattering (SAXS) can also be used to study the formation of zeolites [109,110]. It also has now been applied in combination with microscopy to obtain spatial information about the structural pore properties of zeolite ZSM-5 upon desilication [111].

Vibrational spectroscopic techniques are very commonly used to characterize zeolites. By far the most frequently used vibrational spectroscopic technique to study zeolites is infrared (IR) spectroscopy as it is, among the techniques, the easiest to use and because it can provide information about multiple zeolite features. Besides its possibilities to probe several introduced heteroatoms into the zeolite framework, it is tremendously useful for the characterization of acid sites in zeolites [10]. Solely IR spectroscopy can already be used to study the acidic properties of the materials but developments in the use of probe molecules have vastly increased the possibilities of this technique. When studying the acidity, probe molecules of a basic nature are often used as they can bind to the acid sites in zeolites. Examples of frequently used probes in this field are CO, pyridine and acetonitrile [10,112–115]. In this way, Mintova and colleagues used probe molecules in IR and NMR spectroscopy to prove that Mo atoms were incorporated into the MFI structure [113]. Additionally, applying different probe molecules that differ in size and nature could, besides the information about the acidity, also give information about diffusion. IR spectroscopy can also be combined with microscopy and thereby provide spatial information [116]. A downside of IR spectroscopy is that water is strongly absorbing in this region, limiting the analysis of, for instance, the zeolite framework and the influence of the synthesis procedure and ion exchange. The interference of water with the Raman scattering process is relatively low and therefore Raman spectra can be used to study a zeolite framework stretching between 1200 and 700 cm^–1^ [10]. Therefore, zeolites with the same topology but different elemental compositions and exchanged cations can be investigated [117]. Furthermore, Raman spectroscopy, especially when combined with an excitation source in the UV area to induce the resonance Raman effect, can be applied *insitu* or even *operando* to follow solid catalyst changes or hydrocarbon products and intermediates, and is further discussed in ‘Zeolite characterization under working conditions: *operando* spectroscopy’ Section [117].

NS methods for zeolite characterization are another type of vibrational spectroscopic technique applied to study zeolite properties [13]. For instance, inelastic neutron scattering (INS) is based on vibrational spectroscopy principles and is complementary to other vibrational spectroscopic techniques, allowing the analysis of adsorbed species (e.g. hydrocarbon molecules) in zeolite structures that are not detectable with the more conventional IR and Raman spectroscopic methods. Quasi-elastic neutron scattering (QENS) is mainly used to follow hydrogen-containing molecules inside inorganic materials like zeolites, making it possible to follow the hydrocarbon-transport processes [13,12,118]. Novel developments using these techniques involve their use under real working conditions and will therefore be further discussed in ‘Zeolite characterization under working conditions: operando spectroscopy’ Section.

Because of the ion-exchange capabilities of zeolites, the study of exchanged or incorporated metals in the zeolite framework has been of great importance to their applications. EPR is, among other techniques, capable of determining the nature and the environment of some introduced metals such as iron, nickel, cobalt, manganese and copper by using the magnetic character of the elements [14,15,119–121]. An example of a very recent study extending the use of EPR as a key technique together with DFT calculations has been performed by Chiesa and colleagues. They used H_2_^17^O to introduce the magnetic isotope of oxygen to be able to study the metal–oxygen bonds in Cu-CHA zeolites to allow further elaboration on the structure–property relations in these materials [122].

As mentioned for many studies above, during the discovery of numerous properties of zeolites, characterization techniques often make use of molecular simulations and calculations to support the spectroscopic data (e.g. DFT calculations, Rietveld refinement and other computational spectra predictions) and to form general consensuses. These calculations and models are of great importance as support for zeolite characterization and vice versa. Especially under circumstances that cannot (yet) be detected due to sensitivity issues or to catalyst and/or (intermediate) product stability, molecular simulations and calculations are extremely valuable. Obviously, many other great examples of combining calculations with spectroscopic data exist and the following examples are just used to point out some very recent important work. Chizallet and co-workers combined NMR and Fourier-transform infrared (FTIR) spectroscopy with periodic boundary DFT calculations. The DFT calculations served as a key tool in this investigation to obtain further information on the surface OH groups on zeolite ZSM-5 depending on the crystallite size [123]. Additionally, Dib *et al.* applied a similar combination of techniques to study the effect of silanol groups in silicalite-1 [82]. NS techniques are also often used in combination with molecular simulations. De Leeuw and co-workers used QENS together with molecular dynamic simulations to further elucidate the role of phenolic monomer in the conversion of lignin in zeolite Beta [124]. The synergy between modeling and experimental research will stay extremely important for the revelation of many structure–property relations in zeolite catalysts and there is no doubt that these approaches will go hand in hand in the near and far future.

## ZEOLITE CHARACTERIZATION UNDER WORKING CONDITIONS: *OPERANDO* SPECTROSCOPY

One of the most important industrial applications of zeolites is their use in catalysis, in which these materials are functioning as shape-selective solid catalysts for many crucial reactions. Many of these reaction mechanisms are extremely complicated and hard to study under ambient conditions as they are changing very quickly. Therefore, spectroscopic insights into zeolite properties under non-ambient conditions (*insitu*) and the working behavior of zeolites and the reaction products are extremely useful for unraveling the complicated mechanisms and to further optimize catalyst design. In the last 20 years, many characterization techniques, from the more conventional to the more novel techniques, have been developed to work under industrially relevant conditions and when such experiments are combined with actual product tracing by methods such as online mass spectrometry (MS) or gas chromatography (GC), we refer to it as *operando* conditions. These *operando* conditions allow the correlation of catalytic performance with physical changes of the zeolite or can provide information about the reaction intermediates and deactivating species. In this part, we would like to highlight some important developments in *insitu* and/or *operando* spectroscopy and microscopy. Again, some techniques need high-intensity radiation sources, while other techniques can be used on the lab scale. Several techniques are used to obtain spatial information of the zeolite and its reaction products, while other methods are used to obtain information about the bulk by looking into the light interaction of the zeolite material and/or the interaction with the reaction intermediates. Therefore, this section will be divided in three parts focusing on (i) chemical imaging, (ii) light–intermediates/product interactions and (iii) beam–catalyst interactions.

### Chemical imaging of zeolites under working conditions

To obtain spatial information of zeolites under working conditions, microscope techniques are often used as they can map the catalyst activation and deactivation. For many of these techniques, a hard restriction is the zeolite crystal size as otherwise no high-resolution and thereby proper quality data can be obtained. Therefore, often large zeolite model catalysts are used. A reaction frequently studied by means of *operando* and *insitu* imaging is the MTH reaction as it is easy to perform using mild conditions (ambient pressures and temperatures between 300°C and 500°C), which allows the use of reaction cells with a transparent quartz window. Additionally, the reaction intermediates of this reaction are olefinic and aromatic hydrocarbons, which absorb light of different wavelengths depending on their nature, e.g. alkylation, double bonds and the number of aromatic rings.

**Figure 9.**
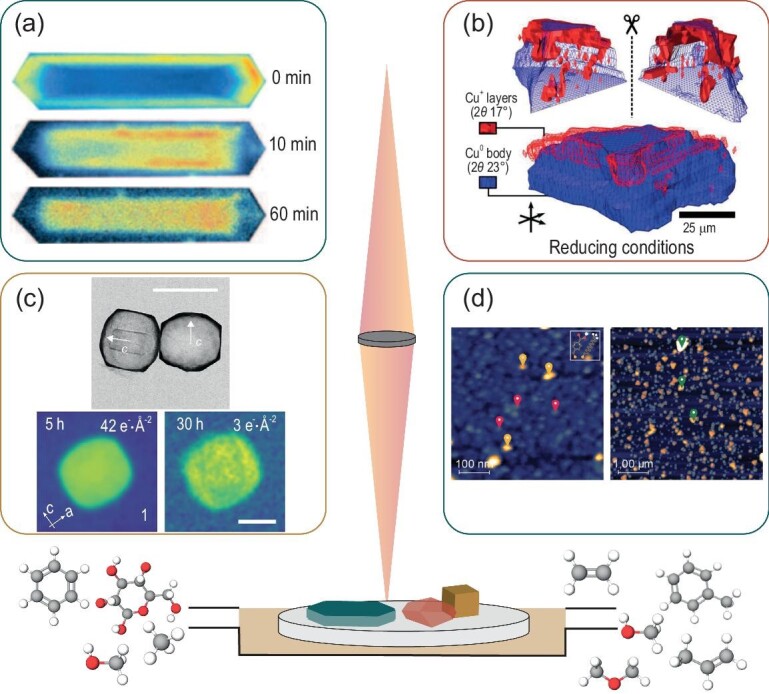
Examples of imaging (microscopy) techniques used to study inorganic porous materials under working conditions. (a) Confocal fluorescence microscopy (CFM) images of HZSM-5 crystals during the MTH reaction at 387°C after 0, 10 and 60 min time-on-stream (TOS), measured using a 561-nm laser in a detection range of 565–635 nm. Images obtained with permission from Ref. [32], Copyright (2008) Wiley-VCH. (b) 3D render of the hard X-ray tomography data with a Cu^0^ core and a Cu_2_O phase at the core–shell interphase of zeolite ZSM-5. Image reprinted with permission from Ref. [127], Copyright (2017) American Chemical Society. (c) Scanning electron microscopy (SEM) images of ZSM-5 crystals and liquid-phase transmission electron microscopy (LP-TEM) imaging of the desilication of single-particle ZSM-5 crystals over time illustrating the proof of concept that EM can be used to study zeolites under working conditions in liquid phase. Scale bar is 400 nm. Figure adapted with permission from Ref. [128], Copyright (2021) American Chemical Society. (d) AFM images of the MMBA-UDT surface and HKUST-1 to demonstrate the application of *in situ* PiFM on metal organic framework (MOF) materials to highlight the possibilities for further investigations in zeolite chemistry. Figures obtained with permission from Ref. [129], Wiley-VCH.

As mentioned earlier, (aromatic) probe molecules can be used to study the zeolite properties using CFM. Important reaction intermediates and products in many hydrocarbon-containing reactions, like MTH, are aromatic molecules and can therefore directly be used as probe molecules. Mores *et al.* used CFM and UV-vis microscopy to spatially resolve the hydrocarbon reaction intermediates in zeolite ZSM-5 crystals (Fig. 9a) and SAPO-34 stressing their coking behavior differences on the microscale (single crystal). Different reaction intermediates for the different zeolite frameworks were observed [32]. The difference in the nature of the crystal and framework structure also led to clear differences in spatial coking/deactivating behavior. They found that the formation of deactivating coke molecules in ZSM-5 begins at the external surface of the crystals, after which it gradually proceeds into the crystal, which is slowed down at the intergrowth structure boundaries resulting a visible hour-glass pattern at certain laser excitation wavelengths. Conversely, the formation of coke molecules in the corners and edges of SAPO-34 crystals prevents the further diffusion of reactants and products to the inner crystal core [32]. The influence of water on the MTH reaction has also been studied with both CFM and UV-vis microscopy in combination with molecular simulations, showing a slower and more homogenous discoloration (slower formation of coke molecules) of the SAPO-34 catalyst [125]. Moreover, not only the MTH reaction can be studied with these techniques; many other reactions involving hydrocarbon conversions can be studied in this manner, such as the aromatization of paraffins and olefins [126].

X-ray-based imaging techniques, like STXM and μ-XRD, can also be applied to study zeolite materials under working conditions. Sheppard *et al.* combined multiple synchrotron-based X-ray imaging techniques with each other to study and form a 3D image of the catalyst Cu/ZnO/Al_2_O_3_@ZSM-5 (core@shell) for the formation of DME from syngas via methanol. Due to the clear core@shell structure of the catalyst particles, it was discovered that the zeolite shell had an important influence on the Cu-catalyst state while preserving the core–shell structure of the catalyst [127]. Figure 9b shows a 3D rendered image of the Cu core at the core–shell interface under reducing conditions obtained using hard X-ray tomography.

The use of EM under working conditions has been greatly developed in recent years [22]. Although, to the best of our knowledge, the technique has not been applied yet to study zeolites under real gas-phase working conditions (e.g. high temperature or pressures with reactant gasses), methods to study zeolite changes over time have been developed using liquid-phase (LP) EM or liquid-cell (LC) EM. However, the use of such liquid environments limits the *insitu* capabilities of this technique to reactions taking place in liquid phase. Recently, such *insitu* liquid-phase experiments were performed by Patterson and colleagues and the desilication process of ZSM-5 zeolites was visualized [128]. Some of these images are depicted in Fig. 9c. Gas-phase TEM to study catalysts while they were fed with certain gasses has been developed but not yet applied to zeolites [22]. We therefore like to emphasize this technique to be very promising in further investigations in the zeolite research field as it ideally could provide further knowledge of the zeolite structure and morphology upon reaction and regeneration.

Another imaging technique that we believe would be very promising for studying zeolite materials and their working mechanisms in different process is *operando* and/or *insitu* PiFM. *Insitu* PiFM has been proven to be very useful for studying molecule adsorption and desorption in porous materials such as metal–organic frameworks (Fig. 9d) [129]. This characterization technique is only surface-sensitive, allowing the further understanding of the surface reactions on zeolite crystals. Using a similar approach, applying TERS as an *insitu* technique for studying zeolite surfaces is also a promising field as it has already been demonstrated for the study of surfaces in other processes, e.g. photo- and electrochemical processes [130,131].

### Reaction intermediate identification by spectroscopy

**Figure 10.**
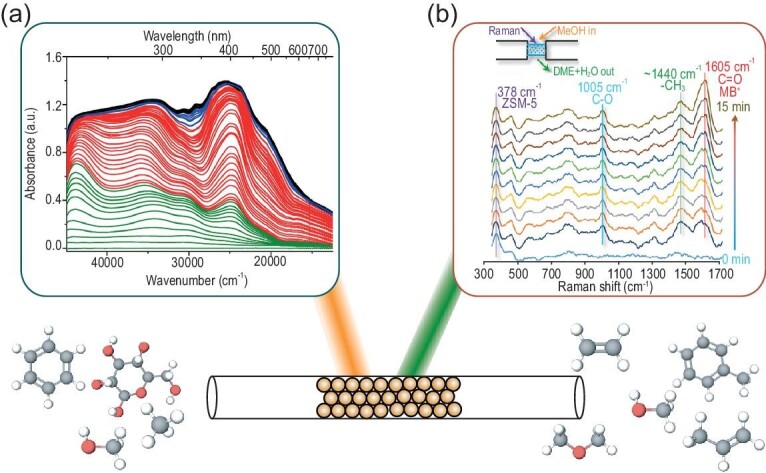
Examples of *operando* spectroscopy to follow the conversion of methanol under working conditions. (a) *Operando* UV-vis spectra of the MTH reaction over SAPO-34 at 400°C showing several bands at different wavelengths corresponding to active hydrocarbon pool species and deactivating poly-aromatic coke molecules. Figure reprinted with permission from Ref. [132], Copyright (2017) American Chemical Society. (b) UV-Raman spectra obtained in the first 15 min of the methanol-to-DME reaction using ZSM-5 as a catalyst at the beginning of the reactor bed at 250°C. Figure reprinted with permission from Ref. [137], Copyright (2018) American Chemical Society.

*Operando* UV-vis spectroscopy can be used to study a range of intermediate products within zeolites during reactions, such as the MTH process. As previously mentioned, aromatic molecules act as active intermediates as well as deactivating compounds and some of them can interact with light in the UV-vis wavelength range. This allows the analysis of the formation and the contribution of these arenes to the activation and deactivation period for multiple reactions for which coke molecules are the deactivating or activating compounds. This technique has been used by our group to study the temperature dependency of the CHA and MFI structure in the MTH process, the effect of feedstock impurities, the influence of the zeolite crystal structure in small-pore zeolites and the influence of the reaction bed position (Fig. 10a depicts an example of UV-vis spectra during the MTH reaction over SAPO-34) [132–135].

*Operando* Raman spectroscopy has also been used by different research groups to study zeolites under working conditions. Nevertheless, to be applicable for this purpose, some problems concerning overlapping fluorescence signals, sample damage and long exposure times had to be overcome. Recently, UV-Raman spectroscopy has proven to be very efficient to study the hydrocarbon intermediates and residues in zeolites with its resistance to the interruption of fluorescence while keeping its high sensitivity due to the resonance enhancement effect [136–138]. An *et al.* were able to identify key intermediate (i.e. methylbenzenium carbenium ion) molecules in the methanol dehydration process by using isotope-switching experiments, allowing the identification of the deactivation and activation phases [137]. Figure 10b depicts one of the UV-Raman spectra taken over the first minutes of the reaction in the beginning of the catalyst bed. At the same time, the group of Zonghmin found that, by partially regenerating the zeolite materials after the MTH process, the selectivity to ethylene was enhanced by the direct transformation of the coke molecules to naphtalenic molecules. This was studied using a wide variety of techniques, among which was *operando* UV-Raman spectroscopy [139]. An interesting alternative to UV-Raman spectroscopy that can also circumvent the interruption of fluorescence is Kerr-gated Raman spectroscopy in which the Kerr-gated spectrometer can differentiate between the Raman signal and the fluorescence signal. With this technique, Beale and co-workers also studied the MTH process over zeolites. Together with molecular simulations, they have established the importance of polyenes in this reaction and their role in the deactivation of the zeolite catalyst during the MTH reaction process [140].

NS analysis techniques were also recently found to be usable under *insitu* and *operando* conditions, especially as they can provide complementary information to UV-vis and Raman spectroscopy. Multiple groups have combined this technique with other *insitu* characterization methods to obtain further insights into catalytic processes. Lin *et al.* studied the reaction of γ-valerolactone (for biomass conversion) to butene over hetero-atomic MFI-type zeolite NbAIS-1. *Insitu* INS, combined with DFT calculations, SXRD and XAS, contributed to the better understanding of the mechanisms within this process [141]. Similarly, the same group contributed to the further understanding of the MTH process by establishing a catalytic cycle over an MFI structure with Ta(V) centers incorporated [142].

Applications for which *insitu*/*operando* IR spectroscopy is useful and widely studied is the NH_3_/NOx–SCR reaction, cracking reactions, conversion reactions and in photocatalysis [10]. Recently published work gave new insights into the SCR reaction mechanism of NO with NH_3_ (NH_3_–SCR) over Cu–AFX using *operando* IR spectroscopy, combined with *operando* UV-vis and XANES spectroscopy and DFT calculations [143]. *Operando* IR spectroscopy can also be used to discover the intrinsic rate and thermodynamic parameters as recently demonstrated by Kadam *et al.* where they applied this technique to the alkane-cracking process [144].

NMR spectroscopy can also applied as an *insitu*/*operando* analysis method to discover reaction mechanisms. Reactions containing hydrocarbon molecules can be followed by isotopically labeling the carbon atom (^13^C-NMR). In this way, the methanol and ethanol conversion over zeolites can be followed as well as the solvent-mediated adsorption reaction of carbohydrates (glucoses), phenol alkylation and the conversion of cyclohexane in water into zeolites [145–149].

Recently, van Bokhoven and co-workers applied a multi-*operando* analysis technique approach in which they combined UV-vis, EPR, XAS and FTIR to identify the active sites and to study the kinetic characteristics in the Cu–MOR catalysts active in the methane-to-methanol conversion. With these techniques, they were able to identify the state of the Cu atoms in the zeolite structure and define their contributions to catalysis [150].

### Catalyst-properties analysis upon activation and deactivation

Due to their use in catalytic reactions, the zeolite catalyst can change in its composition and structure during use. It is interesting to observe whether these changes contribute to catalyst activation and deactivation, and therefore methods to analyse the structural or compositional changes over time are developed.

XAS has been widely applied in an *operando* setting as this technique was discovered early on to provide information under working conditions. One important recent study was performed by Imbao *et al.* Together with kinetic studies, XAS(XANES) was used to study the ethylene oxidation reaction and the influence of water on the Pd–Cu/ZSM-5 and Pd–Cu/Zeolite Y catalysts. In this way, the authors were able to identify the oxidation states and environment of the metals present in the zeolite during the reaction [151,152].

Under catalysis conditions, structural changes in the zeolite framework can occur, which can be detected using spectroscopy, microscopy, diffraction and scattering techniques. When the XRD is measured under working conditions by tracking the reaction products, the changes can be related to the reaction stages. In this way, it was found that the lattice of small-pore zeolites expands in certain directions upon MTH progression because of the formation of larger aromatic molecules in these cages [153]. Similar results were found for zeolite ZSM-22 [154]. This *operando* technique was also applied to spatially study the catalyst bed and to reveal the burning-cigar principle of the MTH reaction by correlating activity and structural changes in zeolite ZSM-5 [155].

## CONCLUDING REMARKS AND PERSPECTIVES

Zeolites are one of the most-studied inorganic porous functional materials as they are used in numerous industrial applications. The detailed characterization of these materials has been performed and studied for 50–100 years, meaning that an enormous amount of literature on this topic exists. The ability of zeolites to be functional in so many applications due to their unique porous stable acidic network leads to the continuing search for answers around zeolite chemistry. This explains the ongoing desire to invent and apply new analytical techniques next to refurbish more conventional analysis methods to unravel these complex but fascinating materials. This review has focused on the most recently developed novel as well as more conventional analytical techniques with the addition of new *operando* spectroscopic insights into their working behavior. The recently developed and used methods (i.e. APT, STXM, PiFM and CFM) are mainly focused on the micro- to nanoscale chemical imaging of the zeolite crystals and the structure and composition of their frameworks. These techniques have contributed to the better understanding of zeolite structures and their performance in a wide variety of applications. Some other analytical techniques do not originate from the recent decade, but have undergone some important further developments, which holds for NMR, EM, IR, Raman, NS and XRD, also allowing better understanding of the properties of zeolites, including the unraveling of their structures and identification of the catalytically active sites. A new development of this century is the characterization of zeolites under catalytic working conditions, allowing simultaneous analysis of the reaction intermediates, products and related material changes over time. This so-called *operando* approach has contributed to the further understanding of the dynamics of zeolite-based catalyst materials. Furthermore, the development of theoretical chemistry approaches has also helped in the identification of e.g. spectroscopic fingerprinting of *operando* data obtained.

Zeolite analysis by means of spectroscopic and characterization techniques has besides great opportunities also some yet inevitable limitations. Some crucial reactions or key chemical elements occur in such low concentrations that they are undetectable with some main analysis methods. However, increasing beam power, to increase sensitivity, can result in beam damage destroying the materials and giving conflicting results. Visualization of the industrially used nanosized zeolite materials with common light microscopes to obtain spatial information is now rather difficult as the resolution of these methods is too low. Increased microscope resolution would predictably lead to further increased knowledge about these materials as, nowadays, large zeolite crystals have to be applied in microscope experiments as model catalysts. Additionally, the elements of which the zeolite materials are composed poses limited z-contrast differences that do challenge the use of many spectroscopic techniques. In general, the limitations mainly involve the sensitivity on spatial and temporal scale for which, in the coming years, proper solutions should be designed. Certainly, the synergy between experimental zeolite characterization and theoretical calculations will be of great importance to bridge this technological gap.

Finally, as spatial, temporal and chemical imaging of zeolite-based materials at the nanoscale is still rather difficult, research evolving around nanoscale structure–composition–performance relations, e.g. between zeolite framework elements and, for instance, reaction products or ions, exchanged on the acid sites, could enhance the understanding of these materials enormously but is still in its infancy. Additionally, further research in the use of other relevant probe molecules to study zeolite chemistry, also in combination with nanoscale spatially resolved analytical techniques, would be highly beneficial.
